# Effects of Temperature on Development and Voltinism of *Chaetodactylus krombeini* (Acari: Chaetodactylidae): Implications for Climate Change Impacts

**DOI:** 10.1371/journal.pone.0161319

**Published:** 2016-08-17

**Authors:** Jeong Joon Ahn, Youngsoo Son, Yaqian He, Eungul Lee, Yong-Lak Park

**Affiliations:** 1 Entomology Program, Division of Plant and Soil Sciences, West Virginia University, Morgantown, West Virginia, 26506, United States of America; 2 Research Institute of Climate Change and Agriculture, NIHHS, RDA, 281 Ayeonno, Jeju, 63240, Republic of Korea; 3 Pierce’s Disease Control Program, California Department of Food and Agriculture, Arvin, California, 93203, United States of America; 4 Department of Geology and Geography, West Virginia University, Morgantown, West Virginia, 26506, United States of America; Universidade Federal de Vicosa, BRAZIL

## Abstract

Temperature plays an important role in the growth and development of arthropods, and thus the current trend of climate change will alter their biology and species distribution. We used *Chaetodactylus krombeini* (Acari: Chaetodactylidae), a cleptoparasitic mite associated with *Osmia* bees (Hymenoptera: Megachilidae), as a model organism to investigate how temperature affects the development and voltinism of *C*. *krombeini* in the eastern United States. The effects of temperature on the stage-specific development of *C*. *krombeini* were determined at seven constant temperatures (16.1, 20.2, 24.1, 27.5, 30.0, 32.4 and 37.8°C). Parameters for stage-specific development, such as threshold temperatures and thermal constant, were determined by using empirical models. Results of this study showed that *C*. *krombeini* eggs developed successfully to adult at all temperatures tested except 37.8°C. The nonlinear and linear empirical models were applied to describe quantitatively the relationship between temperature and development of each *C*. *krombeini* stage. The nonlinear Lactin model estimated optimal temperatures as 31.4, 32.9, 32.6 and 32.5°C for egg, larva, nymph, and egg to adult, respectively. In the linear model, the lower threshold temperatures were estimated to be 9.9, 14.7, 13.0 and 12.4°C for egg, larva, nymph, and egg to adult, respectively. The thermal constant for each stage completion were 61.5, 28.1, 64.8 and 171.1 degree days for egg, larva, nymph, and egg to adult, respectively. Under the future climate scenarios, the number of generations (i.e., voltinism) would increase more likely by 1.5 to 2.0 times by the year of 2100 according to simulation. The findings herein firstly provided comprehensive data on thermal development of *C*. *krombeini* and implications for the management of *C*. *krombeini* populations under global warming were discussed.

*Scientific Article No. 3278 of the West Virginia Agricultural and Forestry Experiment Station, Morgantown, West Virginia

## Introduction

Climate change affected population dynamics of arthropods in natural and agricultural systems such as temporal asynchrony of interacting populations [1,2]. The Coupled Model Intercomparison Project Phase 5 (CMIP5) predicted that significant future climate change and variability would cause severe impacts on various ecosystems. Among various events caused by climate change, temperature increase could be one of the most influential drivers of ecosystem function [3]. The global average surface temperature has increased over the 20^th^ century by ca. 0.6°C [4] and the rate of increasing surface temperature from 1895 through 2013 was about 0.72°C in the contiguous 48 states of the United States [4].

Temperature is a key abiotic environmental factor that influences growth, development, survival and reproduction of poikilothermic arthropods including insects and mites [5–10]. Although some social insects (e.g. honey bees) can decrease the temperature of their nests by fanning their wings, most of arthropods do not have the ability to manipulate temperature within their nests, making them more vulnerable to temperature increase. Arthropod development occurs within a limited range of temperatures and the thermal range varies among arthropod species. Various empirical models have been applied to describe the relationship between temperature and developmental parameters including the lower and upper developmental thresholds and optimal temperatures [11–14]. In addition, developmental parameters often have been applied for optimizing mass rearing systems, predicting phenology, modeling population growth, determining geographical distribution, and simulating climate change impact [15–18]. Various studies already showed that development of arthropods is sensitive to temperature changes [5,19,20] and a small change in temperature could result in spatial and temporal changes in their phenology.

Based on the future climate scenarios of Representative Concentration Pathways (RCPs) [21], annual mean surface temperatures in the eastern United States are predicted with the greatest increase from 2006 to 2100 under RCP 8.5 scenario (+5.7°C), followed by RCP 6.0 (+4.1°C), RCP 4.5 (+3.2°C), and RCP 2.6 (+1.2°C) scenarios (S1 Fig). These four RCP scenarios are new ones presented in the Intergovernmental Panel on Climate Change (IPCC) Fifth Assessment Report (AR5) and provide time-dependent projections of atmospheric greenhouse gas concentrations [21]. The simulated increases in surface air temperature over the eastern United States during the 21^st^ century would be significant enough to impact growth and development of arthropods based on temperature increase in the United States during the last century (< +1°C).

The Krombein’s hairy-fingered mite, *Chaetodactylus krombeini* (Acari: Chaetodactylidae), is a cleptoparasitic mite of *Osmia* bees (Hymenoptera: Megachilidae) which are major or supplementary bees for orchard pollination. *Osmia* bees collect pollen in the field and provision in the nest to feed their larvae. The mite steals and consumes the pollen (i.e. cleptoparasitic), which causes the decline of *Osmia* bee populations [22]. At high density of over 50 adults per cell in an *Osmia* nest [23], *C*. *krombeini* can kill the bee eggs [24] and larvae [22] by attacking and consuming directly. Even if the bee eggs and larvae survive under heavy infestation of the mites within the same cell, the adult bees suffer from reduced fecundity [25] and their pollination efficiency is reduced because the bees get irritated by the mites on their body. Therefore, among the pests of *Osmia* bees, *C*. *krombeini* is known as a key pest that affects the maintenance and propagation of *Osmia* bees for orchard pollination in the United States [22,26,27]. If the mite infestation is not controlled in an orchard, *Osmia* populations could decrease to half from previous year’s size [28].

Developmental stages of *C*. *krombeini* life cycle include egg, larval, protonymph, deutonymph, tritonymph, and adult stages. *Chaetodactylus krombeini* overwinters in *Osmia* bee nests as a deutonymph in two different morphs: encysted and phoretic morphs [23]. Major cures to trigger overwintering of *C*. *krombeini* have not been revealed yet although daylight length, temperature, and food availability could be potential factors. The encysted deutonymphs remain in the same nest over winter until some environmental queues trigger their activity [24]. In spring when temperature increases, the phoretic deutonymphs become active and disperse from old nests to new nests primarily by hitch-hiking on *Osmia* adults [26]. Upon arrival into a new nest of the bee, the phoretic deutonymphs become adult females after molting and each female lays a single egg which develops into a male [24]. Then, a typical life cycle (i.e. egg, larva, protonymph, tritonymph and adult) is repeated and the mites build up their population throughout summer. Later in the season when temperature decreases or pollen provisions are scarce in the nest, protonymphs become either phoretic or encysted deutonymphs for overwintering [24,29].

Despite the economic importance of *C*. *krombeini* as a key pest of *Osmia* bees, little is known about the effect of temperature on the developmental biology of *C*. *krombeini*. This mite does not have any temperature regulation system in its body or behavior adaptation, which makes it an ideal organism to measure the impact of temperature change. This study was conducted (1) to investigate stage-specific development of *C*. *krombeini* at constant temperatures, (2) to develop empirical models to describe thermal development of *C*. *krombeini*, and (3) to predict the spatial and temporal changes in voltinism (i.e., the number of generations) of *C*. *krombeini* under the future climate scenarios.

## Materials and Methods

### Mite colony

*Chaetodactylus krombeini* was collected from the nests of the *Osmia cornifrons* (Hymenoptera: Megachilidae) colony on the West Virginia University Organic Farm in Morgantown, West Virginia (USA) with permission by the WVU Experiment Station. To establish laboratory colonies of *C*. *krombeini*, adult mites were isolated from bee nests and transferred into wells of ELISA plates (E&K Scientific, Santa Clara, CA) mimicking *Osmia* bee nests. Pollen provisions collected from the bee nests in the field were supplied into the wells as a food source for mites. The plates with mite colonies were kept in a 1,000-cm^3^ cardboard box to provide darkness and kept under 20 ± 2°C and 60 ± 10% RH.

### Experimental procedure

Newly-laid *C*. *krombeini* eggs (<1 day old) were collected randomly from the laboratory colonies to create seven sets of mite colonies for experiments. In each set, 50–100 eggs were individually transferred into an ELISA well provisioned with pollen collected from *O*. *cornifrons* nests. The seven sets of mite colonies were placed into environmental chambers (Percival Scientific, Perry, IA; Fisher Scientific, Dubuque, IA) with seven constant temperatures (i.e. 16.1±0.28°C, 20.2±0.73°C, 24.1±0.06°C, 27.5±0.33°C, 30.0±0.08°C, 32.4±0.51°C, and 37.8±0.48°C). Temperature inside each environmental chamber was recorded with a HOBO data logger (HOBO^®^ onset^®^ UX 100 Temp/RH, Cape Cod, MA). Developmental stage (i.e., egg, larva and nymph) of each *C*. *krombeini* was checked and recorded daily. A stereomicroscope (Olympus SZ-ST, Tokyo, Japan) was used to distinguish life stages of *C*. *krombeini* based on size, shape, and quiescence periods between two stages (i.e., larva to nymph and nymph to adult). The adult stage was identified based on the presence of seta on abdomen and sclerotized structure underneath the abdomen [30]. Checking and recording the developmental stages of *C*. *krombeini* needed to take *C*. *krombeini* development out of the chambers. To minimize the potential effect of temperature changes on *C*. *krombeini* development, recording developmental stages was done within 10 min. under 20±0.5°C, 60±10.2% RH.

### Developmental distribution model

The effect of temperature on the duration of development was examined by using analysis of variance (ANOVA) [31]. Regression analyses were used to model temperature-dependent development of each *C*. *krombeini* stage and the model parameters were obtained by using TableCurve 2D Automated Curve Fitting program [32].

Cumulative frequency distribution (%) of stage-specific emergence was plotted against development time (d) at each temperature. The relationship between time and the frequency was described by the cumulative Weibull model [33,34]:

$$f\left(x\right)\;=\;100–100\;\times \text{ exp }\left[-{\left(x/\alpha \right)}^{\beta }\right],$$

where *f*(*x*) is the cumulative frequency distribution (%) at cohort age *x* and *α* and *β* are scale and shape parameters, respectively. The median development time (i.e., time to 50% cumulative frequency) was calculated as *α* × [–ln (0.5)]^1/*β*^. To generate a temperature-independent distribution model for each stage, the cumulative frequency (%) was plotted against the normalized time which was calculated by dividing the development time by the median development time at each temperature (i.e., days / median). The normalized data were pooled across temperatures and fit to the Weibull model [35].

### Development rate model

Development rate at each temperature was calculated as the reciprocal of median development time (1 / median) in days, which was obtained by the Weibull distribution model at each temperature. Over the entire thermal range, the relationship between temperature and the development rate was described by the nonlinear Lactin model [12], which was modified from Logan type I model [13]:

$$R\left(T\right)\;=\text{ exp }\left(\rho T\right)-\text{exp }[\rho T_{L}-(T_{L}-T)\;/\Delta ],$$

where *R*(*T*) is the rate of development at temperature *T*; *ρ* can be interpreted as a composite Q_10_ value for enzyme-catalyzed biochemical reactions; *T*_*L*_ is the lethal maximum temperature; Δ is the width of the decline phase in development rate above the optimum temperature [13]. Optimum temperature (*T*_*opt*_), at which the mite develops at its maximal rate, was calculated using the estimates of model parameters as *T*_*opt*_ = *T*_*L*_*−* Δ. The thermal range with more than 80% performance (*B*_*80*_) was also determined [36]. The 3-parameter Lactin model was selected because it consistently provided significant fits to the temperature-dependent rate data for all *C*. *krombeini* stages, in contrast to alternative 4-parameter nonlinear models.

Data at mid-range of the Lactin model were used to develop a linear model: *y = a + bx*, where *y* is the rate of development at temperature *x*; *a* is the *y*-intercept; and *b* is the slope. Using the parameter estimates, the lower developmental threshold (LDT) was calculated as—(*a* / *b*) and the thermal constant in degree-days (DD) was calculated as 1 / *b* [37].

### Simulation of *C*. *krombeini* adult emergence

Assuming that a cohort of *C*. *krombeini* eggs were continuously exposed to constant-temperature condition, adult emergence in terms of daily frequency (%) was simulated in relation to the temperature (°C) and the time (day) by incorporating two models of *C*. *krombeini* development: the Weibull model for development frequency distribution and the Lactin model for the temperature dependent rate [5,35]. The Lactin model determines the daily rate of development at a given temperature and the Weibull model determines the cumulative frequency (%) at a given temperature and time. Therefore, the mathematical expression of the simulation is:

$$F\left(x,T\right)\;=\;100-100\;\times \text{ exp}\left\{-{\left[x\;\times \;R\left(T\right)\;/\alpha \right]}^{\beta }\right\},$$

where *F*(*x*, *T*) is the cumulative frequency (%) of the *C*. *krombeini* adult emergence at time *x* and constant temperature *T*; *R*(*T*) is the nonlinear Lactin model for the temperature-dependent development of *C*. *krombeini* (egg to adult); *α* and *β* are the scale and shape parameters estimated of the Weibull model against normalized time (egg to adult). From the simulated results in the cumulative frequency (%), the emergence of *C*. *krombeini* adults in daily frequency between time *x* and *x* +1 was calculated by subtracting the cumulative percentage of the cohort at time *x* from that at time *x* + 1.

### Modeling voltinism of *C*. *krombeini*

Determining the number of generations (i.e., voltinism) obtained based on the climate data and developmental parameters, is useful to estimate the establishment potential for invasive species or introduced biological control agents [16,38]. To estimate the voltinism of *C*. *krombeini*, the Kearneysville Fruit Tree Research and Education Center (KFTREC) of West Virginia University located in Kearneysville, West Virginia (USA) (39° 23’ 45” N, 77° 53’ 44”; 160 m in elevation) was selected as a site for modeling and simulation of voltinism. The center had been utilized *Osmia* bees for orchard pollination and experienced problems associated with *C*. *krombeini* infestation during the past ten years. In addition, historical weather data (e.g., hourly temperature) directly collected at the center were available. The developmental parameters estimated herein were applied to calculate degree-days using daily minimum and maximum temperature data of ambient air from weather stations at KFTREC. Specifically, we used the lower development threshold (LDT) and upper developmental threshold (UDT) of 12.4°C and 37.8°C, respectively, for *C*. *krombeini* development from eggs to adults found in this study. Degree-days were calculated by using single sine/vertical cutoff methods [39] and cumulative degree-days (CDD) were obtained by summing calculated degree-days over a period of time. The voltinism of *C*. *krombeini* was calculated by dividing CDD per specific period (month or year) by the thermal constant requirement of *C*. *krombeini* development from eggs to adults.

In addition, the voltinism of *C*. *krombeini* was estimated based on two different types of biofix for degree-day accumulation each year (i.e., determining the first date for degree-day accumulation): one with calendar date of January 1^st^ and the other with the predicted date for the emergence of *O*. *cornifrons* adults in spring. *Osmia cornifrons* adults generally emerge in April in West Virginia [20] and phoretic deutonymphs of *C*. *krombeini* need to move to a new bee nest by hitchhiking on newly-emerging *Osmia* bees. Therefore, the biofix using the predicted date of spring emergence for *O*. *cornifrons* adults could reflect more realistic life cycle of *C*. *krombeini* in the field. Spring emergence date of *O*. *cornifrons* adults was predicted by calculating the degree-day accumulation up to 126.1 DD with LDT and UDT of 10.5°C and 46.2°C, respectively, which were reported in a previous study on *O*. *cornifrons* [20]. Degree-day calculations were done with single sine/vertical cutoff methods [39]. After obtaining the predicted date of *O*. *cornifrons* adult emergence as a biofix, the degree-days for *C*. *krombeini* development were accumulated.

### Projected voltinism of *C*. *krombeini* under the future climate scenarios

The projected voltinism of *C*. *krombeini* throughout the eastern United States (32–48°N and 66–87°W) was calculated by using the developmental parameters (i.e., LDT and UDT) of *C*. *krombeini* found in this study and the future temperature data for 2006–2100 based on various RCP scenarios: RCP 8.5, RCP 6.0, RCP 4.5, and RCP 2.6. Daily minimum and maximum surface temperatures were obtained from the coupled simulations of the Community Earth System Model version 1 using the Community Atmosphere Model version 5 (CESM1-CAM5) [40] under the four RCP scenarios. Based on the simulated model outputs covering 192 (latitude) by 288 (longitude) grid cells (i.e., 0.9375° by 1.25°) for 2006–2100, daily mean temperature was calculated by averaging the daily minimum and maximum temperatures.

**Fig 1:**
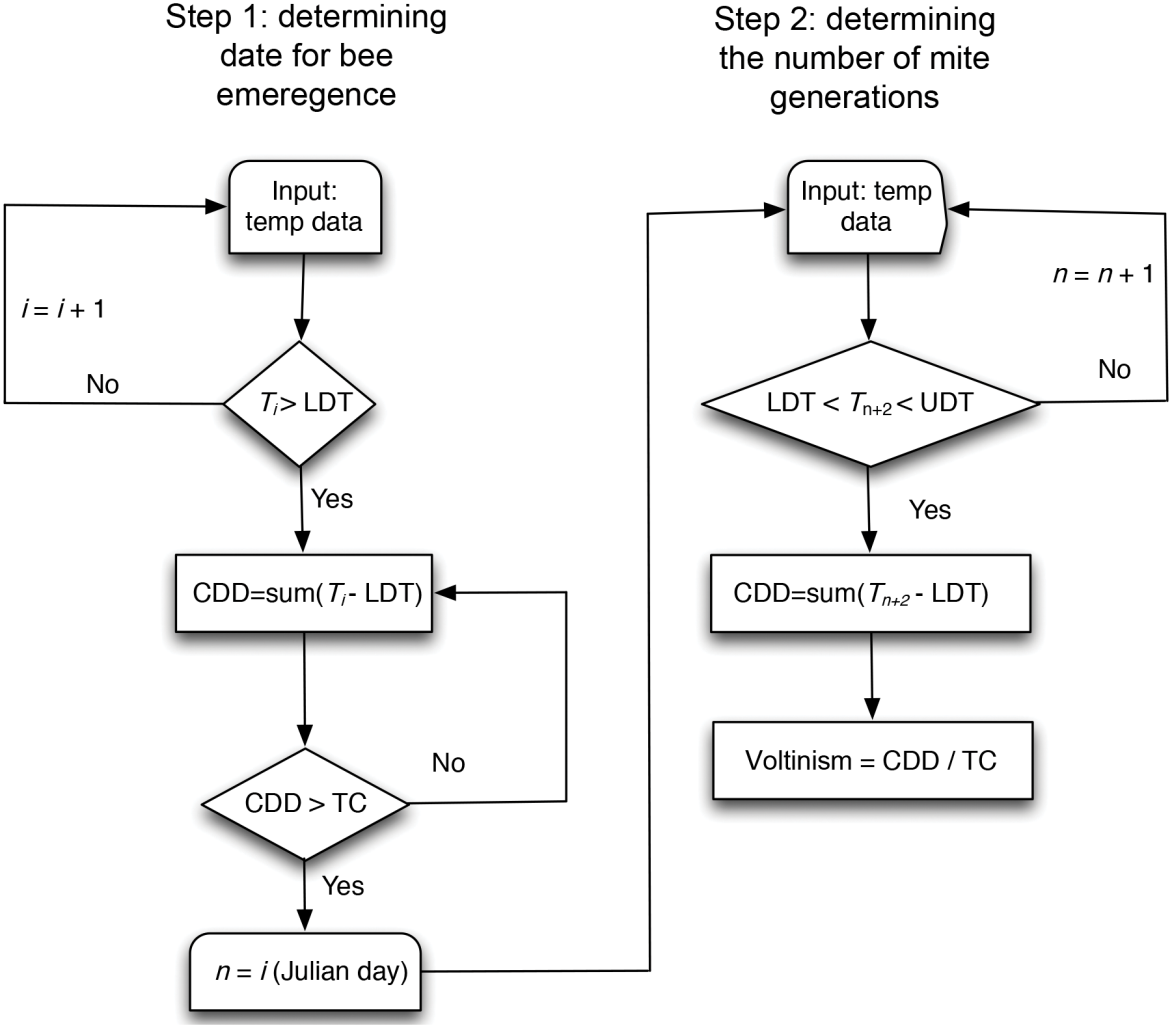
Flowchart for calculating the voltinism of *C*. *krombeini* by using the daily mean surface air temperature under the future climate scenarios. *T*_*i*_, temperature at *i*-th date; LDT, lower temperature threshold; CDD, cumulated degree days; UDT, upper temperature threshold; TC, thermal constant.

To calculate the voltinism of *C*. *krombeini* in each grid cell over the eastern United States from 2016 to 2100, the daily mean surface air temperatures from the coupled CESM1-CAM5 simulations were used and two steps of calculations (i.e., bee emergence and *C*. *krombeini* development) were implemented (Fig 1). First, because *C*. *krombeini* starts its development after *Osmia* bees emerge so that it can hitchhike the bees to move to a new bee nest for colonization and development, the Julian day of bee emergence was required to be determined first. The LDT and thermal constant for *O*. *cornifrons* were set as 8.6°C and 179.8 DD, respectively, which were reported in White et al. [20]. Degree-days were calculated by subtracting LDT from daily mean air temperature and used to obtain CDD for the bee emergence in each grid cell of the eastern United States. No accumulation of degree-days was made in days when daily mean air temperature was lower than LDT. Dates for the bee emergence in spring were obtained by determining the date when CDD was greater than or equal to thermal constant. Second, to calculate the voltinism of *C*. *krombeini*, LDT and thermal constant found in this study were used. Because it takes ca. two days for hitchhikes bees, move to a new nest, and lay eggs, the CDD for *C*. *krombeini* was calculated by adding two days into the Julian day of bee emergence (i.e., *n* + 2 in Fig 1). The voltinism of *C*. *krombeini* was determined by dividing CDD by thermal constant of *C*. *krombeini*. The calculated number of generations per year in each grid cell was displayed over the eastern United States based on the four RCP scenarios. A series of maps for the voltinism of *C*. *krombeini* were generated for the years of 2015, 2050, and 2100. In addition, the differences in the voltinism between 2015 and 2100 under different future climate scenarios were calculated to estimate the spatio-temporal changes in *C*. *krombeini* voltinism.

To investigate the temporal variability in *C*. *krombeini* voltinism, a linear regression analysis was conducted by using the time series of the projected number of generations from 2006 to 2100. The number of generations per year in each grid cell over the eastern United States for the period of 2006–2100 was calculated, and then averaged the over the grid cells corresponding West Virginia (37–41°N and 77–83°W) and its surrounding regions. The trend of time series was analyzed with a regression analysis and the significance of the trend was tested by a Student’s *t* test [31]. The same procedure described above was applied for all the four RCP scenarios.

## Results

Results are presented herein as means ± SEM, unless otherwise noted. *Chaetodactylus krombeini* developed from egg to the adult at all tested temperatures except the highest temperature (37.8°C), where no eggs hatched. The developmental duration of each stage was significantly influenced by temperatures: egg (F_5, 276_ = 107.17, *P* < 0.01), larva (F_5, 182_ = 183.70, *P* < 0. 01), nymph (F_5, 146_ = 70.61, *P* < 0.01), and egg to adult (F_5, 145_ = 241.28, *P* < 0.01) (Table 1). Mean time required for development from egg to adult emergence ranged from 8.8 days at 30.0°C up to 38.2 days at 16.1°C. Cumulative frequency distribution of *C*. *krombeini* development times at each temperature was well described by the Weibull model (*r*^2^ > 0.910, *P* < 0.01) and the median development time was obtained at each temperature (Table 1).

**Table 1:** Development time (days) for *Chaetodactylus krombeini* at constant temperature.

Stage	Temp. (°C)	n	Mean ± SE (day)	Weibull model			
				*α*	*β*	*r*^*2*^	Median (day)
Egg	16.1	35	8.31 ± 0.43	8.43 ± 0.22	3.21 ± 0.31	0.986	7.52
	20.2	54	6.96 ± 0.18	6.60 ± 0.09	9.25 ± 1.36	0.989	6.34
	24.1	66	4.61 ± 0.13	4.35 ± 0.06	6.11 ± 0.58	0.995	4.10
	27.5	48	4.00 ± 0.10	3.75 ± 0.01	6.99 ± 0.12	0.999	3.56
	30.0	41	3.51 ± 0.09	3.21 ± 0.10	5.33 ± 1.20	0.991	2.99
	32.4	36	3.25 ± 0.13	3.04 ± 0.02	4.53 ± 0.18	0.999	2.80
	37.8	0	_^a^	_	_	_	_
Larva	16.1	19	15.58 ± 0.82	15.82 ± 0.17	7.87 ± 1.03	0.983	15.10
	20.2	42	5.29 ± 0.29	4.69 ± 0.16	3.27 ± 0.67	0.985	4.19
	24.1	39	4.49 ± 0.18	4.38 ± 0.06	3.82 ± 0.28	0.996	3.98
	27.5	24	3.25 ± 0.27	2.76 ± 0.09	5.14 ± 1.04	0.985	2.57
	30.0	37	2.16 ± 0.14	1.72 ± 0.05	4.56 ± 0.58	0.997	1.59
	32.4	22	2.23 ± 0.23	1.82 ± 0.02	2.61 ± 0.11	0.999	1.59
	37.8	0	_	_	_	_	_
Nymph	16.1	16	14.94 ± 0.13	15.79 ± 0.68	3.11 ± 0.51	0.911	14.04
	20.2	36	8.67 ± 0.39	9.19 ± 0.16	4.87 ± 0.54	0.980	8.53
	24.1	29	7.48 ± 0.27	7.52 ± 0.07	6.98 ± 0.59	0.995	7.13
	27.5	19	8.53 ± 0 65	9.05 ± 0.18	2.87 ± 0.23	0.983	7.96
	30.0	36	3.19 ± 0.10	2.86 ± 0.02	6.82 ± 0.51	0.999	2.71
	32.4	16	4.19 ± 0.37	3.75 ± 0.07	4.68 ± 0.56	0.994	3.46
	37.8	0	_	_	_	_	_
Egg to adult	16.1	16	38.19 ± 1.77	39.48 ± 0.66	4.94 0.54	0.954	36.65
	20.2	36	20.83 ± 0.48	21.66 ± 0.19	11.31 1.47	0.972	20.97
	24.1	29	16.41 ± 0.29	16.52 ± 0.12	11.77 1.22	0.988	16.01
	27.5	19	15.63 ± 0.60	16.16 ± 0.19	5.93 0.58	0.980	15.19
	30.0	36	8.83 ± 0.15	8.48 ± 0.05	16.30 1.50	0.997	8.30
	32.4	16	9.38 ± 0.58	8.82 ± 0.17	6.98 1.24	0.981	8.37
	37.8	0	_	_	_	_	_

^a^ No individual survived.

### Developmental distribution model

**Fig 2:**
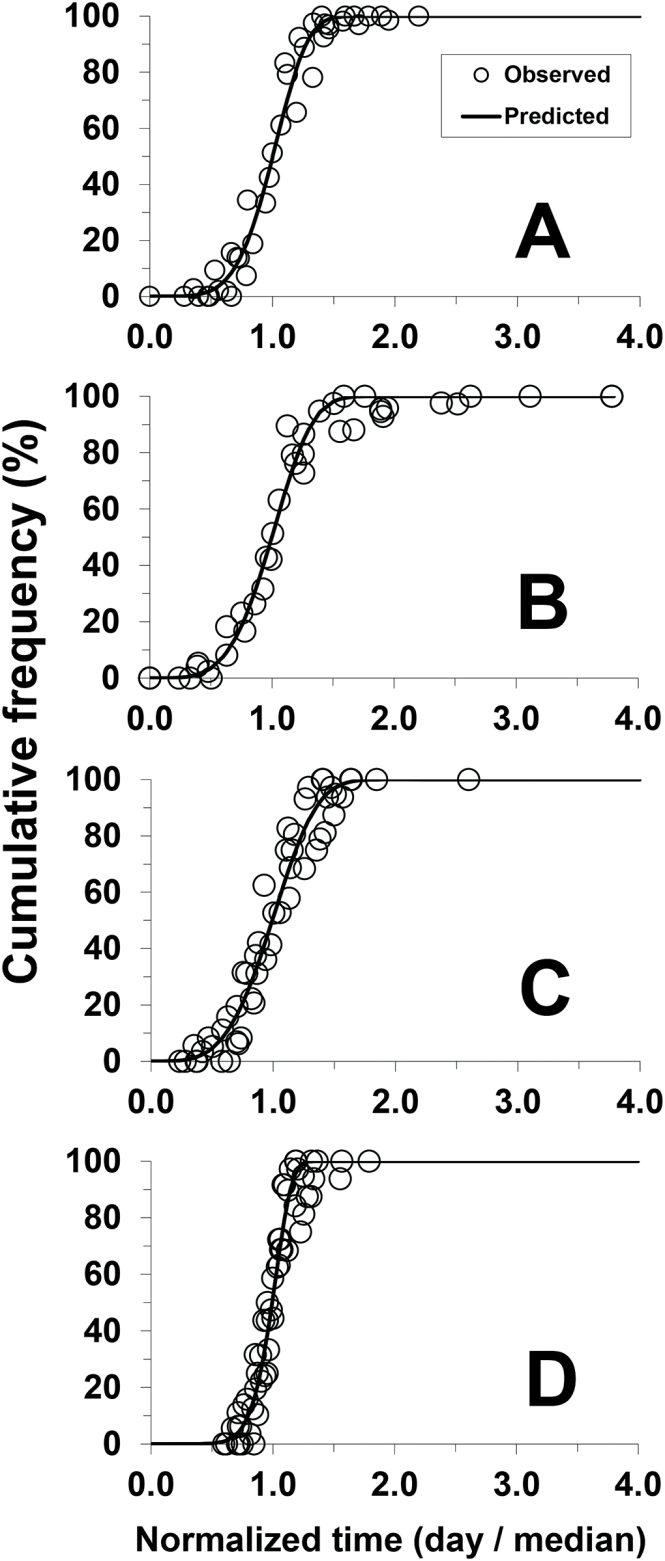
Cumulative frequency distribution (%) of *C*. *krombeini* development against the normalized time (day / median), fit to the Weibull model. A: egg, B: larva, C: nymph, and D: egg to adult.

Against the normalized time (days / median), the stage-specific frequency distribution of *C*. *krombeini* was also described by the Weibull model: egg (F_1,39_ = 1563.9, *P* < 0.01, *r*^2^ = 0.976), larva (F_1,40_ = 1847.5, *P* < 0.01, *r*^2^ = 0.979), nymph (F_1,50_ = 992.0, *P* < 0.01, *r*^2^ = 0.953), and egg to adult (F_1,54_ = 935.2, *P* < 0.01, *r*^2^ = 0.946) (Table 2, Fig 2).

**Table 2:** Parameter estimates (± SE) of the Weibull distribution models for *Chaetodactylus krombeini* development against the normalized time (day / median).

Stage	Parameter	Estimate ± SE	*r*^2^
Egg	*α*	1.079 ± 0.013	0.976
	*β*	5.009 ± 0.382	
Larva	*α*	1.088 ± 0.013	0.979
	*β*	4.275 ± 0.325	
Nymph	*α*	1.089 ± 0.015	0.953
	*β*	3.912 ± 0.272	
Egg to adult	*α*	1.038 ± 0.007	0.946
	*β*	8.518 ± 0.642	

**Fig 3:**
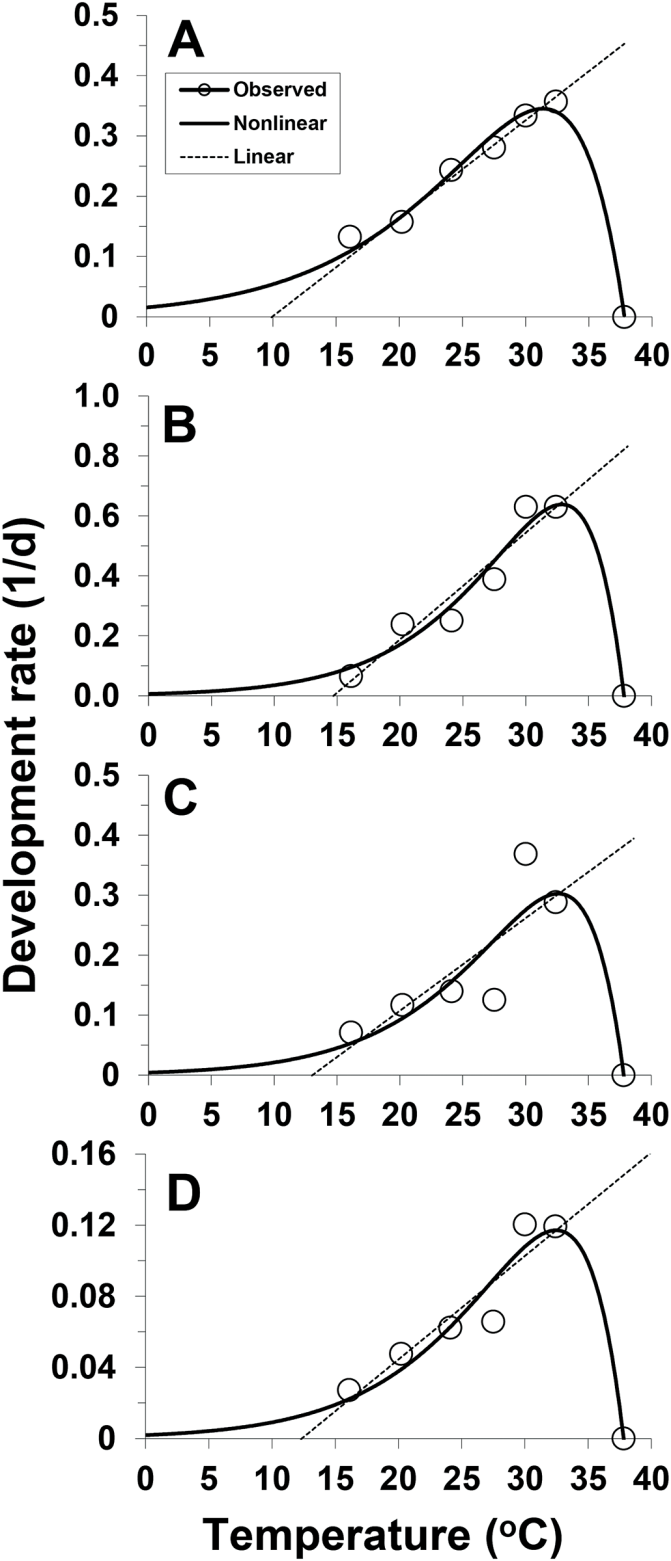
Nonlinear and linear development models for temperature-dependent rates (1 / days) of *C*. *krombeini* development. A: egg, B: larva, C: nymph, and D: egg to adult.

For all the stages, temperature-dependent pattern of *C*. *krombeini* development rate over the entire range showed a typical skewed-bell shape, with sharp decline of the rates at high temperatures above the optimal temperature (Fig 3). The nonlinear Lactin model provided significant fit to the temperature-dependent rate of *C*. *krombeini* development: egg (F_3,6_ = 125.4, *P* < 0.01, *r*^2^ = 0.984), larva (F_3,6_ = 47.2, *P* < 0.01, *r*^2^ = 0.959), nymph (F_3,6_ = 7.8, *P* < 0.05, *r*^2^ = 0.795), and egg to adult (F_3,6_ = 27.4, *P* < 0.01, *r*^2^ = 0.932) (Table 3, Fig 3). The stage-specific optimal temperatures (*T*_*opt*_) with the maximal rate were estimated to be 31.4, 32.9, 32.6 and 32.5°C for egg, larva, nymph, and egg to adult, respectively. At the optimal temperatures, the earliest development completion would occur 2.9, 1.6, 3.3 and 8.5 days for egg, larva, nymph, and egg to adult, respectively. The stage-specific thermal ranges (*B*_*80*_) with ≥ 80% of the maximal rates were calculated to be 26.1–34.7°C, 28.8–35.4°C, 28.3–35.3°C and 28.1–35.2°C for egg, larva, nymph, and egg to adult, respectively.

**Table 3:** Parameter estimates (± SE) of the nonlinear and the linear models for *Chaetodactylus krombeini* development.

Stage	Model	Parameter	Estimate ± SE	*r*^2^
Egg	Nonlinear	*ρ*	0.1550 **±** 0.0075	0.984
		*T*_*L*_	37.816**±** 0.134	
		Δ	6.436 **±** 0.309	
	Linear	*a*	-0.16161 **±** 0.3328	0.983
		*b*	0.01626 **±** 0.00122	
Larva	Nonlinear	*ρ*	0.2019 **±** 0.0187	0.959
		*T*_*L*_	37.808 ± 0.177	
		Δ	4.948 ± 0.454	
	Linear	*a*	-0.52289 ± 0.13479	0.920
		*b*	0.03556 ± 0.00525	
Nymph	Nonlinear	*ρ*	0.1918 ± 0.0415	0.795
		*T*_*L*_	37.815 ± 0.449	
		Δ	5.211 ± 1.122	
	Linear	*a*	-0.20111 ± 0.14055	0.665
		*b*	0.01543 ± 0.00548	
Egg to adult	Nonlinear	*ρ*	0.1872 ± 0.0209	0.932
		*T*_*L*_	37.813 ± 0.241	
		*ΔT*	5.339 ± 0.596	
	Linear	*a*	-0.07200 ± 0.02791	0.878
		*b*	0.00582 ± 0.0109	

Linear regression models were fit to the development rate data in the mid-range: egg (F_1,4_ = 176.4, *P* < 0.01, *r*^2^ = 0.983), larva (F_1,5_ = 45.9, *P* < 0.01, *r*^2^ = 0.920), nymph (F_1,5_ = 7.94, *P* < 0.05, *r*^2^ = 0.665), and egg to adult (F_1,4_ = 28.7, *P* < 0.01, *r*^2^ = 0.878) (Table 3, Fig 2). Based on the pattern of the nonlinear Lactin model, the data ranges chosen for linear regression analysis were 20.2–32.4°C for egg and 16.1–32.4°C for all the other stages. The LDTs were estimated to be 9.9, 14.7, 13.0 and 12.4°C for egg, larva, nymph, and egg to adult, respectively. The thermal constant (DD) over the stage-specific LDT were 61.5, 28.1, 64.8 and 171.8 DD for egg, larva, nymph, and egg to adult, respectively.

**Fig 4:**
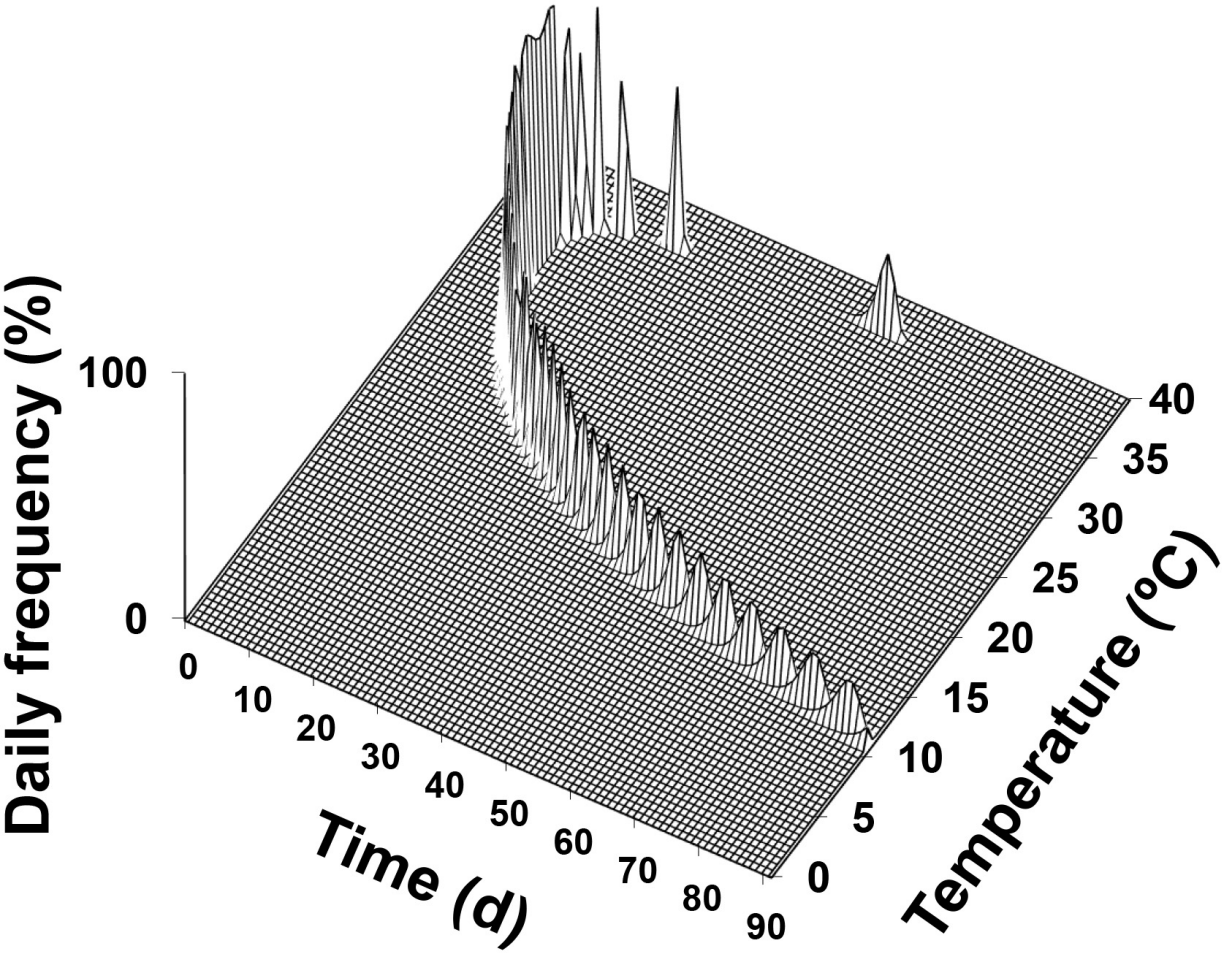
Simulation of daily frequency (%) of *C*. *krombeini* adult emergence from an egg cohort in relation to constant temperature (°C) and time (day).

Given a cohort of *C*. *krombeini* eggs under constant temperatures, the predicted daily frequency (%) of adult emergence was presented in relation to temperature (°C) and time (day) (Fig 4). The predicted values showed that adult emergence of *C*. *krombeini* would occur earlier at the optimum temperature in much shorter time but the adult emergence at both ends of temperatures would occur later over extended periods. From a cohort of *C*. *krombeini* eggs, for instance, the adult emergence would occur in 8–10 days at 34°C, whereas it would take 70–84 days at 12°C and 21–25 days at 37°C.

### Simulation of adult emergence

The mean number of generations per month, estimated from degree-days available per month at KTFREC, was shown in Table 4. We found differences in the degree-days accumulated depending on the calendar months (F_11, 107_ = 276.30, *P* < 0.01) and the number of generations possible for *C*. *krombeini* per month (F_11, 107_ = 248.18, *P* < 0.01). The number of generations highly varied depending on the season, with the lowest (0.01) in February and the highest (2.16) in July. At least one generation per month would be possible during May–September. Based on the biofix of January 1^st^, the voltinism of *C*. *krombeini* ranged from 8.6 to 10.7 generations at KTFREC over the past nine years (Table 5). Similarly, predicted dates of *O*. *cornifrons* emergence in spring varied from March 28^th^ to May 1^st^, depending on the year.

**Table 4:** Estimated seasonal voltinism of *Chaetodactylus krombeini* based on the degree-day accumulation in Kearneysville, West Virginia (USA).

Month	Mean (± SEM) per month	
	Degree-day accumulation^a^	Number of generations^b^
January	5.0 ± 1.6	0.03 ± 0.02
February	4.1 ± 1.4	0.01 ± 0.01
March	30.1 ± 7.3	0.18 ± 0.05
April	83.5 ± 5.1	0.48 ± 0.03
May	181.9 ± 11.0	1.07 ± 0.07
June	296.3 ± 8.1	1.73 ± 0.05
July	369.9 ± 13.4	2.16 ± 0.08
August	332.3 ± 11.9	1.94 ± 0.07
September	213.7 ± 8.2	1.22 ± 0.05
October	100.1 ± 11.8	0.59 ± 0.07
November	21.7 ± 4.2	0.12 ± 0.03
December	5.4 ± 1.5	0.02 ± 0.01

^a^ Degree-days were calculated using the lower and upper thresholds of 12.4 and 37.8°C, respectively, and thermal requirement of 171.1 DD.

^b^ Numbers of generations were estimated from the degree-days per month, calculated from the weather station data from 2006 to 2014.

**Table 5:** Estimated annual voltinism of *Chaetodactylus krombeini* over a 9-year period in Kearneysville, West Virginia (USA), based on different biofix: January 1^st^ and date of spring emergence of *Osmia cornifrons* adults.

Year	Biofix of January 1^st^		Biofix of bee emergence date		
	Thermal constant (DD)	No. generations	Bee emergence date^a^	Thermal constant (DD)	No. generations
2006	1737.7	10.16	14 April	1656.0	9.64
2007	1698.7	9.93	24 April	1613.1	9.39
2008	1470.9	8.60	24 April	1390.6	8.10
2009	1483.7	8.67	25 April	1407.6	8.20
2010	1837.2	10.74	10 April	1745.7	10.17
2011	1708.2	9.98	24 April	1626.7	9.47
2012	1728.6	10.10	28 March	1644.5	9.58
2013	1592.6	9.31	20 April	1503.2	8.75
2014	1537.2	8.98	1 May	1453.6	8.47
Mean ± SEM	1643.9 ± 42.5	9.61 ± 0.25		1560.0 ± 41.6	9.09 ± 0.24

^a^ Spring emergence dates for *Osmia cornifrons* adults were predicted from the degree-day calculation from the same weather data, based on the lower and upper developmental threshold of 10.5 and 46.2°C, respectively, and thermal requirement of 126.1 DD [20].

By using the host emergence date as the biofix each year, the numbers of generations per year were estimated to be less (i.e., 8.1 to 10.1 generations per year), compared to the estimated values using the biofix of January 1^st^. However, the difference was not significant (*P* > 0.05); the small difference appears to have resulted from the lack of degree-days under the low temperatures prevailing at KTFREC during January through April (Table 4).

### Projected voltinism of *C*. *krombeini* under the future climate scenarios

**Fig 5:**
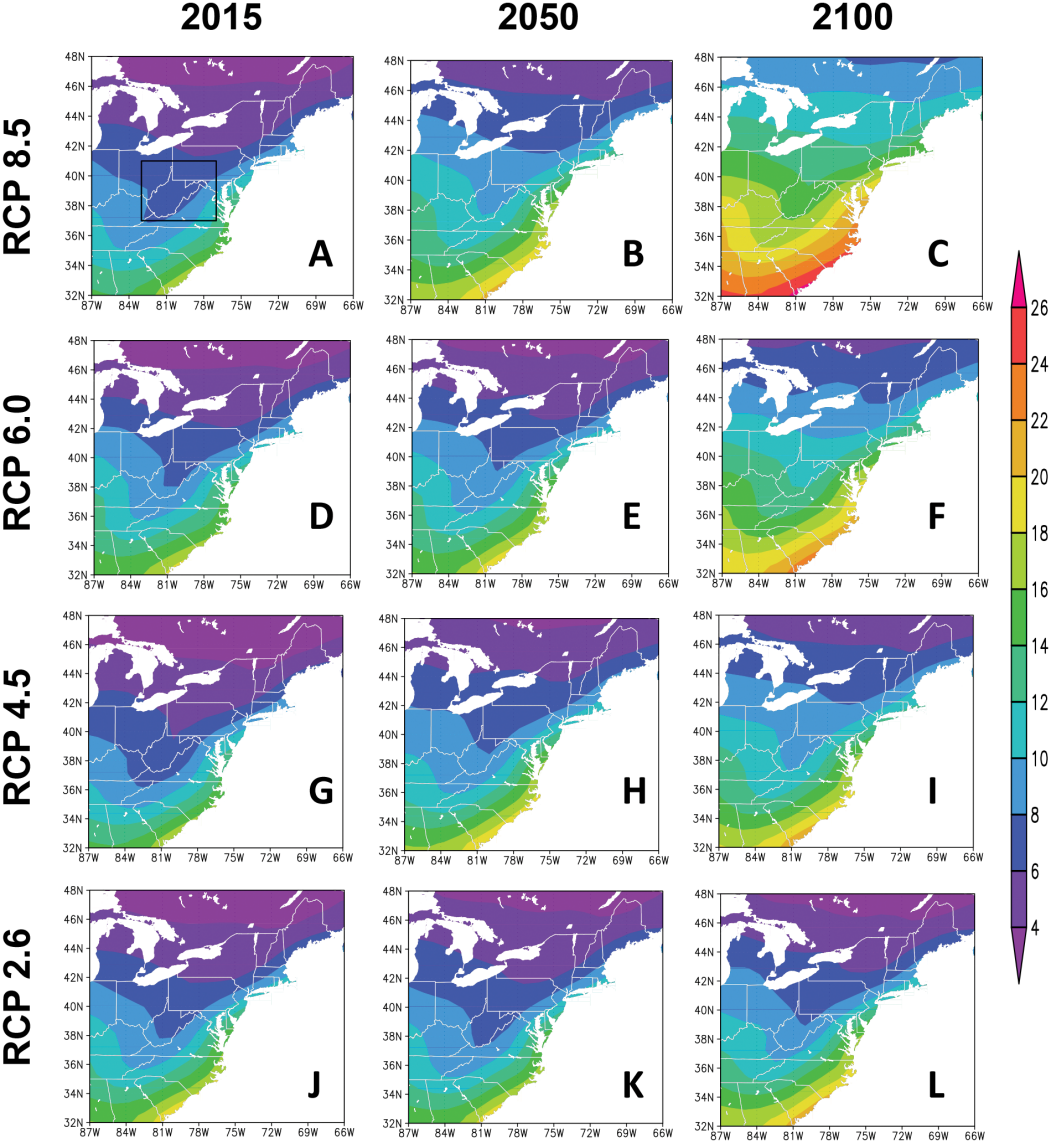
Predicted spatial patterns of *C*. *krombeini* voltinism in the eastern United States in 2006–2100 based on the four climate scenarios of RCP 2.6, RCP 4.5, RCP 6.0, and RCP 8.5.

**Fig 6:**
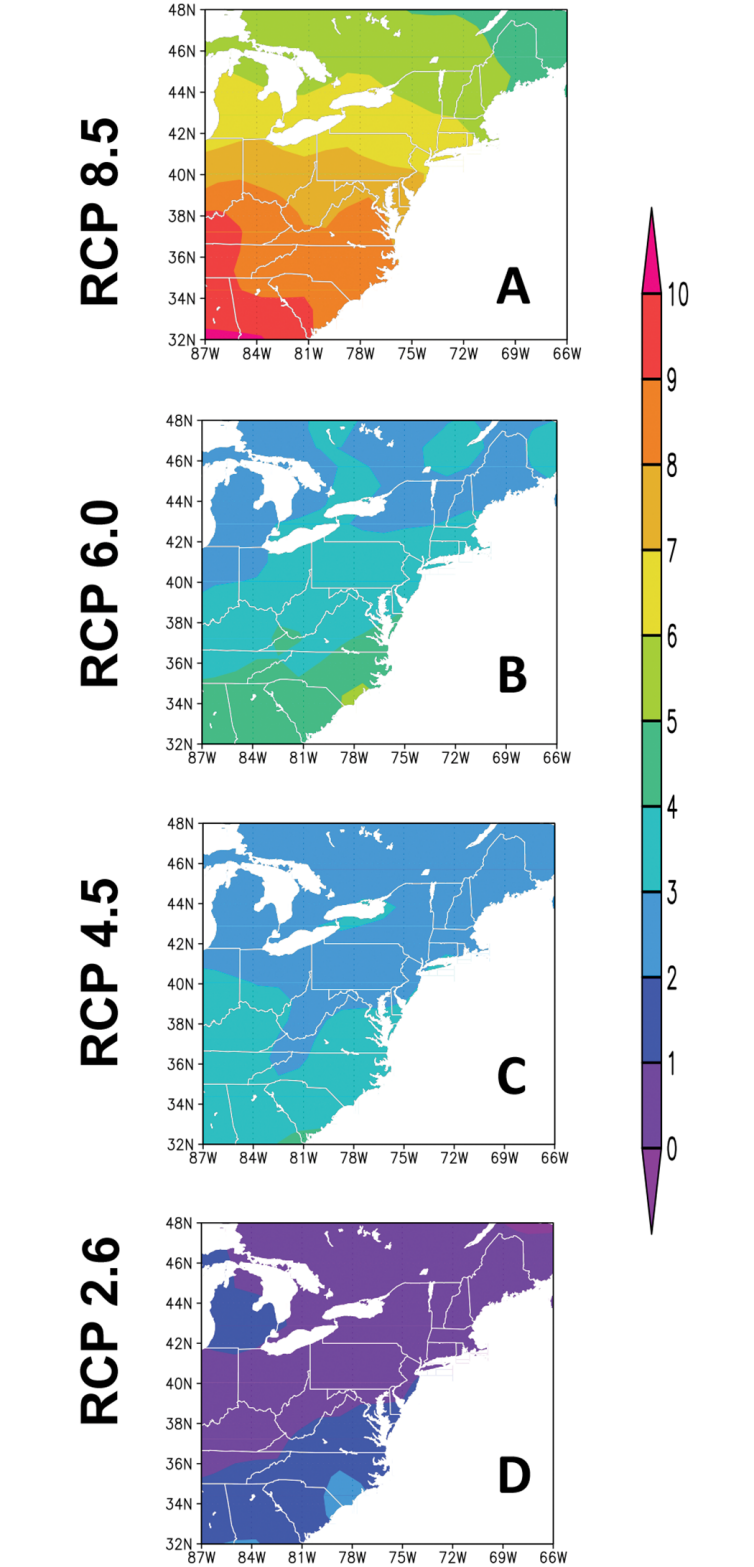
Spatial maps for the increase of the number of *C*. *krombeini* generations in the eastern United States in 2100 relative to 2015 based on the four climate scenarios of RCP 2.6, RCP 4.5, RCP 6.0, and RCP 8.5.

Spatial distributions of projected number of generations of *C*. *krombeini* over the eastern United States showed significant differences (Fig 5) among the years of 2015, 2050, and 2100 under the four RCP scenarios. In 2015, the voltinism from the four RCP scenarios was estimated to be 6–10 generations in the eastern part of West Virginia where KTFREC was located. This estimation of voltinism based on the future climate scenarios was consistent with that from our simulation using two different biofixes at KTFREC. The voltinism of *C*. *krombeini* was generally higher in the lower latitude regions in the eastern United States. In 2015, the voltinism was consistent among the four RCP scenarios with gradual increasing from four to eighteen generations from the northern to southern parts of the eastern United States. The number of *C*. *krombeini* generations increased by 2050 (Fig 5B–5E, 5H and 5K) and more apparent increase was predicted by 2100 (Fig 5C, 5F, 5I and 5L). The rates of changes in *C*. *krombeini* generations in the eastern United States from 2015 to 2100 were different among the four RCP scenarios (Fig 6). The voltinism increased by 0–2 and 4–10 generations per year in RCP 2.6, and RCP 8.5 scenarios, respectively, by the year of 2100 (Fig 6A–6D). The voltinism based on the RCP 4.5 (Fig 6C) and RCP 6.0 (Fig 6B) scenarios were increased by 2–4 and 2–5 generations, respectively, by 2100. In all four climate scenarios, the rate of increase in voltinism of *C*. *krombeini* was higher in the lower latitudes of the eastern United States.

**Fig 7:**
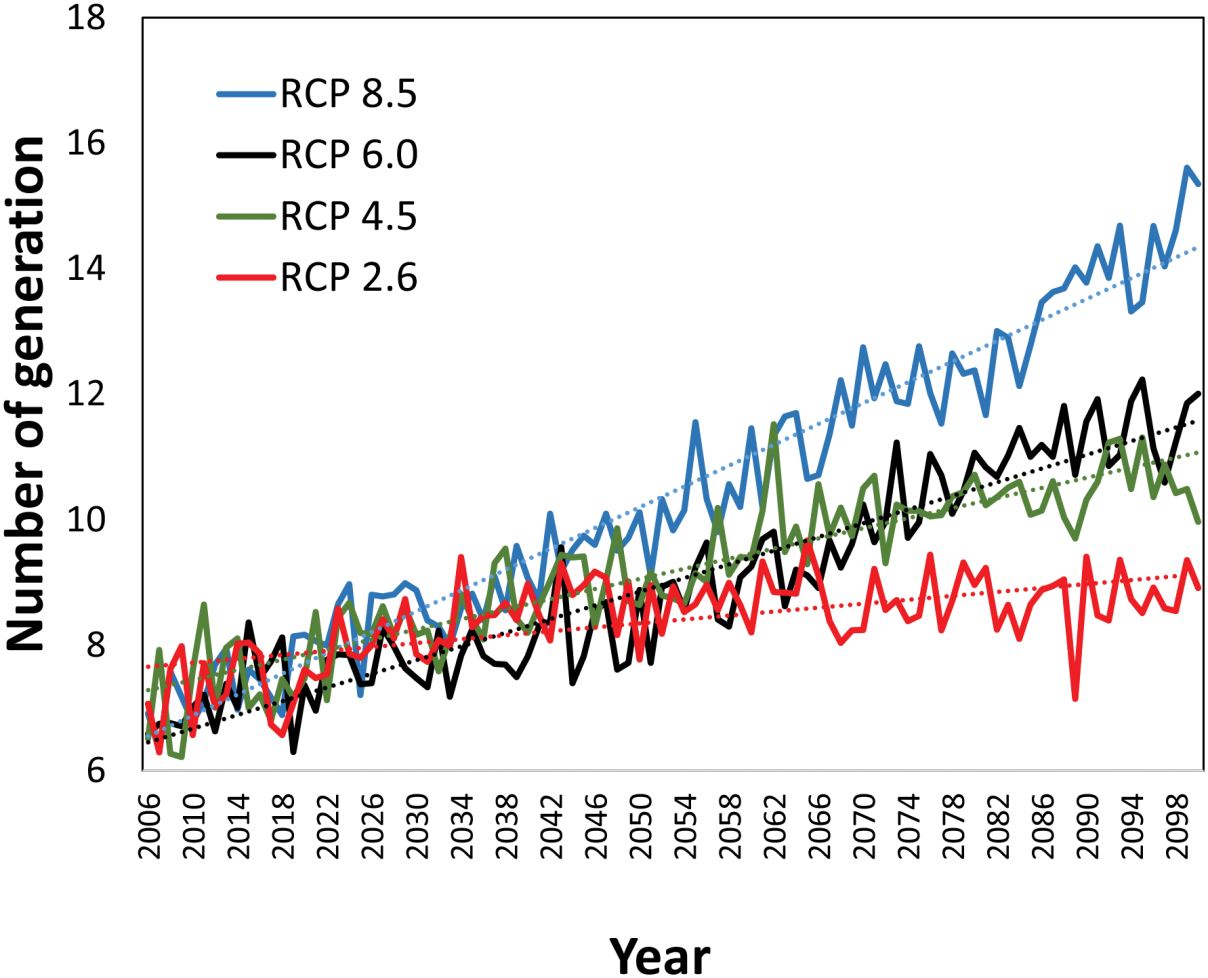
Temporal changes in the voltinism of *C*. *krombeini* in West Virginia and its surrounding regions (37–41°N and 77–83°W; see the boxed area in Fig 5A) in 2006–2100 under the four climate scenarios of RCP 2.6 (*y* = 0.0157*x* + 7.6399, *P* < 0.01), RCP 4.5 (*y* = 0.0403*x* + 7.241, *P* < 0.01), RCP 6.0 (*y* = 0.0545*x* + 6.398, *P* < 0.01), and RCP 8.5 (*y* = 0.0829*x* + 6.466, *P* < 0.01).

To examine the temporal dynamics of the *C*. *krombeini* voltinism under the future climate scenarios, we performed the liner regression analysis by using the time series of the generation number. The number of *C*. *krombeini* generations was estimated to be significantly (*P* < 0.01) increased by 2100 under the four RCP scenarios (Fig 7). The greatest rate of increase in voltinism was 0.83 generations per decade based on the RCP 8.5 over the 95 years from 2006 to 2100. The predicted rates of increase in voltinism based on the RCP 6.0 and 4.5 were 0.55 and 0.40 generation per decade, respectively. In RCP 2.6, the lowest rate of increase in voltinism was 0.16 generation per decade and the time-series trend appeared relatively stationary beyond 2050. The results under the projected climate change indicated that the number of *C*. *krombeini* generations in the central Appalachian regions would increase more likely up to 1.5–2.0 times above their current levels (i.e., 8–10 generations per year) by the end of the 21^st^ century.

## Discussion

This study firstly demonstrated the stage-specific thermal development of *C*. *krombeini* by applying empirical models, which provided developmental parameters crucial to predict phenology and damage of the mite. Development rate models in this study showed that *C*. *krombeini* development from egg to adult emergence would occur within the thermal range of 12.4°C and 37.8°C (i.e., between LDT and UDT) and the fastest growth was observed at 32.5°C (i.e., optimum temperature for development). At the optimum temperature, it would take 8.54 days for the mite egg to develop into adult, which also indicates the maximum number of generations would be possible in a season when the ambient temperature prevails near the optimum temperature or at locations where temperature in regional climate stays longer near the optimum temperature. When an actual field temperature was used to calculate the voltinism more realistically (Table 5), the mite would be able to complete up to 8–10 generations per year in Kearneysville, West Virginia (USA). Seasonal pattern of voltinism at the location showed much variation depending on the months with higher values in summer (May-September), which clearly suggests that the early application of control measures on the infested nest must be considered to prevent the rapid population increase in summer.

In commercial orchards, bundles of nests (e.g. cardboard tubes or reeds) are typically used to facilitate easy maintenance of *Osmia* bees [23,28] including *O*. *cornifrons* and *O*. *lignaria*. Such method, however, provides an ideal habitat for the mites [22,26]. Currently, *C*. *krombeini* is controlled by cultural and chemical methods such as removing bee cocoons from bee nests to remove *C*. *krombeini* [22], cleaning bee cocoons with bleach solution to clean *C*. *krombeini* [22,41], and using essential oils [42] to fumigate bee nests and kill *C*. *krombeini* directly. Alternatively, treating bee nests with very low or high temperatures would negatively affect the survival and population of *C*. *krombeini*. Because *Osmia* bees’ development and survival also depend on temperature [43,44], determining the optimal temperature that maximize both the bee’s survival and maximize the mite’s mortality would be important to use temperature as a physical control method. Results of our study suggest the potential of using high temperature as a control measure against *C*. *krombeini* for *Osmia* bee propagation and maintenance, based on the difference in optimal temperatures and UDTs between the *Osmia* bee and the *C*. *krombeini*. In spring, optimum temperature and UDT of *O*. *cornifrons* adult emergence are 35.7 and 46.2°C, respectively [20], whereas those for *C*. *krombeini* development herein are 32.5 and 37.8°C, respectively. This indicates that *C*. *krombeini* would suffer more than *O*. *cornifrons* if exposed to high temperatures (e.g. 35.7–37.8°C). During summer diapause of *O*. *cornifrons*, this method would be more effective because the diapausing *O*. *cornifrons* becomes tolerant to heat while the mites still remain susceptible to high temperatures [45]. The effects of exposure duration and high-temperature treatment on both *C*. *krombeini* and *O*. *cornifrons* survival have yet to be determined in a future study in order to find out the optimal combination of the exposure duration and the treatment temperature, which can maximize the mortality of *C*. *krombeini* but have little or no impact on *O*. *cornifrons* survival. Moreover, heat treatment may be also considered as a post-treatment option after chemical control, if a certain pesticide in apiculture may have positive temperature-dependent toxicity [46,47].

The voltinism of *C*. *krombeini* estimated with the daily mean surface air temperature from the coupled CESM1-CAM5 simulations were consistent with those from the experimental results in Kearneysville, West Virginia. The spatial patterns from the simulated climate data well captured the latitudinal gradients of *C*. *krombeini* voltinism depicted by the increased number of generations from north to south over the eastern United States. Additionally, the lower numbers of generations in the central and southern Appalachian regions, compared to the surrounding regions in the same latitudes, were generally explained by showing the isolines bending southward in the mountainous regions (Fig 5). Although the climatic data used in this study represented well the overall spatial patterns of the voltinism in the eastern United States with the daily temporal interval and approximately 100 km^2^ spatial resolution, a higher spatial resolution should be beneficial to capture the local and regional changes in the mite generations under the future climate change.

In addition to temperature, water vapor deficit (VPD) can be an important factor that affects *C*. *krombeini* under future climate change. VPD is the difference between saturation vapor pressure and actual vapor pressure [48]. Actual vapor pressure and relative humidity are linearly associated when air temperature is constant and saturation vapor pressure increases exponentially as temperature increases [49]. Therefore, under the condition with increased VPDs at higher temperature, insects and mites can lose higher amount of moisture to the air by evaporation. Although the effect of VPD on *C*. *krombeini* has not been reported, previous studies documented increased mortality of some predatory mites (e.g. *Amblyseius cucumis* and *Neoseiulus califonicus*) at higher VPDs [48,50–55]. We also observed a higher mortality rate of *C*. *krombeini* at higher temperatures in our study, which also could be caused by higher VPDs because relative humidity was kept constant at 60±10% in all growth chambers (i.e. same or very similar actual vapor pressures across chambers). However, no studies have found that VPD could affect the rate of insect or mite development which is a key function in modeling temperature-dependent development of *C*. *krombeini* in our study although effects of VPD on mite development was studied [56]. Cook et al. [57] successfully simulated the increased VPD with the RCP 8.5 scenarios at the global scale, and thus additional studies to investigate the effect of various VPDs on mite development could lead to incorporating VPD in the development model and generating more realistic prediction of future biological events of the mite (e.g. development period and voltinism).

Operative thermal ranges of *C*. *krombeini* and the *Osmia* bee clearly show that the host and the parasite have a discreet thermal sensitivity profile for development [20], indicating that *Osmia* bees and *C*. *krombeini* would respond differently exposed to the same temperature in the field. While the short-term exposure of high temperature is of great interest for the management of *C*. *krombeini* (i.e., sensitivity to high temperature), the relatively small and realistic change of climate in the long-term may alter drastically the host-parasite interaction, geographical distribution, and abundance as shown in other species [15, 58]. Despite sensitivity to high temperature extremes, the mite may acquire tolerance to the extreme by acclimation following non-lethal condition and the slowly-elevated temperature may allow an increase in the voltinism per year due to faster development and longer season available for multiple reproductive phases.

## Conclusion

Results herein provide fundamental information regarding the thermal development of all the life stages of *C*. *krombeini*. *C*. *krombeini* development from egg to adult emergence would occur within the thermal range of 12.4°C and 37.8°C (i.e., between LDT and UDT) and the fastest growth was observed at 32.5°C (i.e., optimum temperature for development). This study also showed how the mite’s voltinism could be affected by not only future temperature increases but also host bees’ temperature-dependent spring emergence which is also affected by the future temperature increase. Temperature can play a major role in altering voltinism of *C*. *krombeini* according to the future climate change scenarios; the number of generations of the mite would increase by 1.5–2.0 times by the year of 2100.

In addition, findings in this study firstly provided comprehensive data on thermal development of *C*. *krombeini* and implications for an effective control and mitigation strategy against *C*. *krombeini* for the propagation and management of *Osmia* bees for pollination. Operative thermal ranges of *C*. *krombeini* and the *Osmia* bee are different and they have a discreet thermal sensitivity profile for development, indicating the potential of using temperature as a method to control *C*. *krombeini*; high-temperature treatment may have potential for *C*. *krombeini* control because the mite is more sensitive to high temperatures than *Osmia* bees. Future studies may be needed to determine the optimal combination of the exposure duration and the treatment temperature.

## Supporting Information

S1 Fig Annual mean surface temperatures in the eastern U.S. from 2006 to 2100 under RCP scenarios. Data were obtained from http://cmip-pcmdi.llnl.gov/cmip5/index.html
(PDF)
