# Supplementary material for: Effects of Temperature on Development and Voltinism of Chaetodactylus krombeini (Acari: Chaetodactylidae): Implications for Climate Change Impacts
Source: PLoS One. 2016 Aug 17;11(8):e0161319. doi: 10.1371/journal.pone.0161319 (PMC4988649; doi:10.1371/journal.pone.0161319)

S1. Annual mean surface temperatures in the eastern U.S. from 2006 to 2100 under RCP scenarios. Data were obtained from http://cmip-pcmdi.llnl.gov/cmip5/index.html

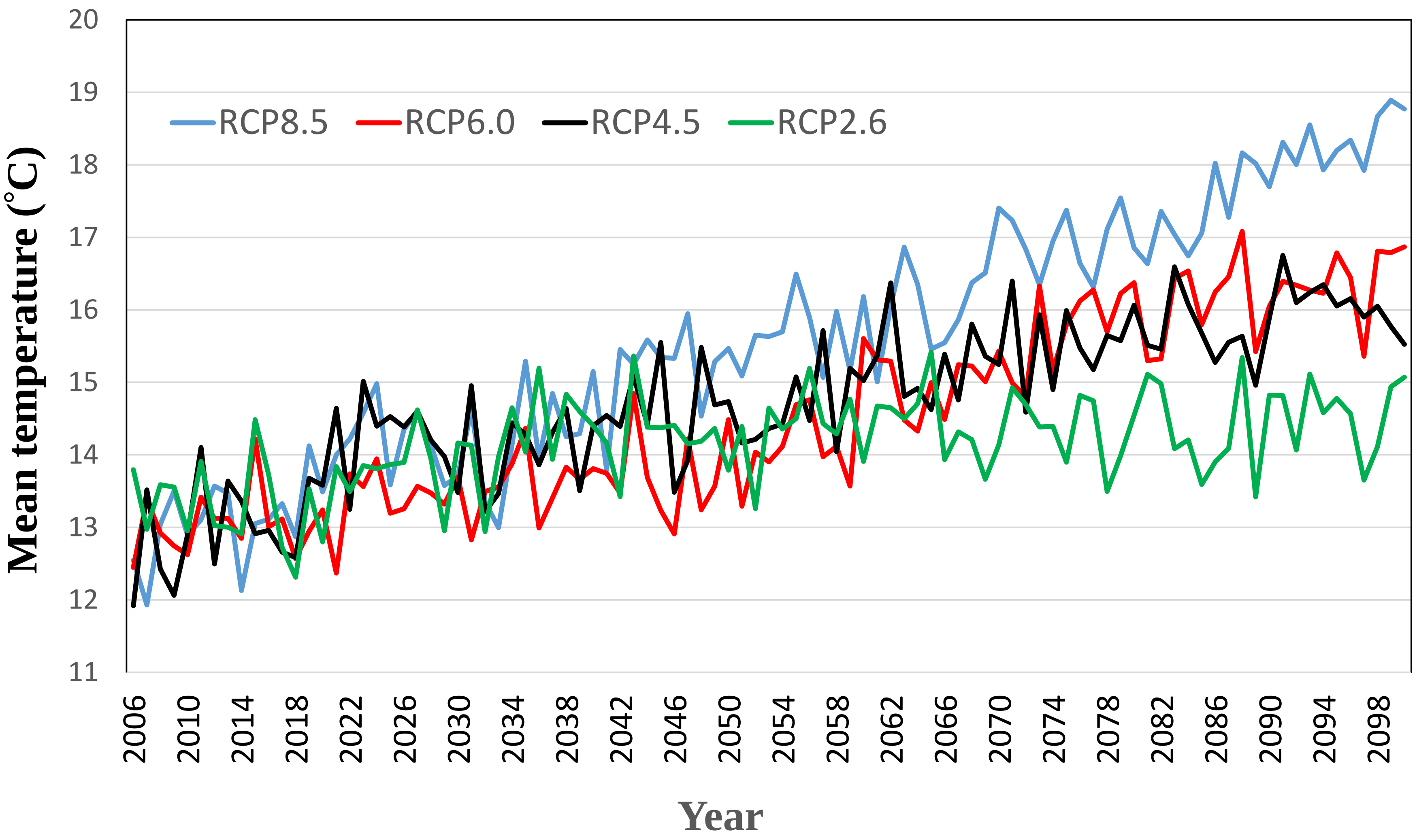

S2. Temperature data used for simulation. Data were obtained from http://www.caf.wvu.edu/kearneysville/weatherstations.htm

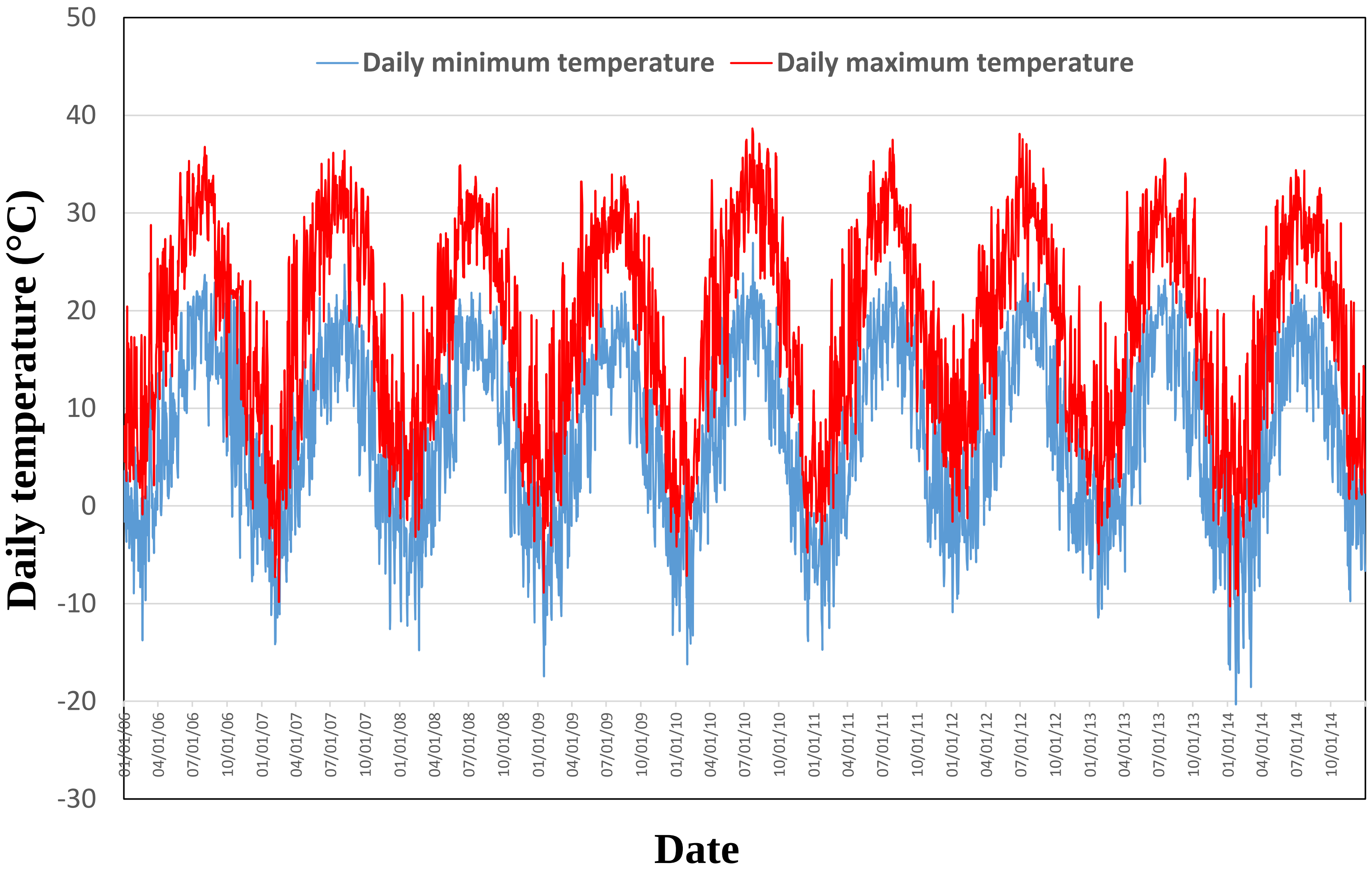

Supplement: S1 Fig — Data were obtained from http://cmip-pcmdi.llnl.gov/cmip5/index.html (PDF) [file pone.0161319.s001.pdf]
